# Omphalocele: national current birth prevalence and survival

**DOI:** 10.1007/s00383-021-04978-z

**Published:** 2021-08-15

**Authors:** Anna Fogelström, Cecilia Caldeman, Jenny Oddsberg, Anna Löf Granström, Carmen Mesas Burgos

**Affiliations:** 1 Division of Pediatric Surgery, Astrid Lindgren Children’s Hospital, Karolinska University Hospital, 17176 Stockholm, Sweden; 2 Department of Women’s and Children’s Health, Karolinska Institute, 17177 Stockholm, Sweden; 3 Department of Surgery, Danderyd Hospital, 18257 Stockholm, Sweden; 4 Department of Clinical Sciences, Danderyd Hospital, Karolinska Institute, 18288 Stockholm, Sweden

**Keywords:** Omphalocele, Exomphalos, Prevalence, Termination of pregnancy, Associated anomalies, Mortality

## Abstract

**Purpose:**

The increase in prenatal diagnosis together with the high rates of associated anomalies in omphalocele has led to increased rates of termination of pregnancies. The aim of this study was to examine the national Swedish birth prevalence and survival rates among these patients.

**Methods:**

This study is based on a nationwide population-based cohort of all children born in Sweden between 1/1/1997 and 31/12/2016. All omphalocele cases were identified though the Swedish National Patient Register and the Swedish Medical Birth Register. Outcome of malformations and deaths were retrieved from the Swedish Birth Defects Register and the Swedish Causes of Death Register.

**Results:**

The study included 207 cases of omphalocele (42% females). The birth prevalence for omphalocele was 1/10,000 live births. About 62% of the cases had associated malformations and/or genetic disorders; most common was ventricular septal defect. The mortality within the first year was 13%. The rate of termination of pregnancy was 59%.

**Conclusion:**

The national birth prevalence for omphalocele in Sweden is 1/10,000 newborn, with high termination rates. Over half of the pregnancies with prenatally diagnosed omphalocele will be terminated. Among those who continue the pregnancy, 1-year survival rates are high.

**Type of study:**

National register study

**Level of evidence:**

III.

## Introduction

Omphalocele is one of the most common congenital abdominal wall defects, although still considered a rare diagnosis. It occurs in 1–3.8/10,000 pregnancies [1–6]. In omphalocele, organs protrude through a midline abdominal wall defect together with the umbilical cord. In contrast to gastroschisis, the organs are covered by a sac, constituted of peritoneum, Wharton’s jelly and amnion. An intact sac can partly protect the organs from exposure to amniotic fluid and the external environment.

The size of the defect differs but also the extent of organs that protrude. Defects larger than 5 cm, containing not only intestines but also most of the liver and sometimes stomach, uterus, ovaries or spleen, are often referred to as giant omphalocele. The exact mechanism for the development of omphalocele is not yet established. There are different theories explaining the failure of mesodermal closure when forming the abdominal wall that leaves a larger defect than the umbilical ring [7].

In high-income countries, 96% of the omphaloceles are identified on routine ultrasound [8]. Sixteen to sixty-nine percent of children born with omphalocele have concurrent anomalies and genetic disorders [6, 9–12] including chromosomal anomalies, e.g., trisomy 13, 18 and 21 and other syndromes like Beckwith Wiedemann Syndrome and Pentalogy of Cantrell. Early miscarriages and intrauterine fetal demise are suspected to be common. In Europe, a considerable number of women carrying a fetus with omphalocele choose to terminate the pregnancy (TOP) after prenatal counseling [6, 8, 9, 11, 13].

An overall mortality of 14–30% has been reported [3, 4, 9, 11, 14]. In cases of isolated omphalocele, survival rate can be as high as 90% [9, 15].

We hypothesized that, due to increased rates of prenatal diagnosis and TOP, the prevalence of newborns with omphalocele would be low. The aim of this study was to explore the current birth prevalence, associated anomalies and survival rates for patients born with omphalocele at a population-based level.

## Materials and methods

### Study design

This study is based on a nationwide population-based cohort of all children born in Sweden between 1st of January 1997 and 31st of December 2016. Data were collected from the Swedish National Patient Register (NPR), The Swedish Medical Birth Register (MBR), the Swedish Birth Defects Register and the Swedish Register of Causes of Death. All cases of omphalocele were identified with the ICD-10 code Q79.2 (Omphalocele/Exomphalos) through NPR and the Swedish Medical Birth Register. To avoid inclusion of misclassified subjects, each case had to satisfy one of the following inclusion criteria:

1. Omphalocele as the main diagnosis and a surgical intervention code specific for omphalocele.
2. Admission to a pediatric surgical center at least once with an in hospital stay of at least seven days and omphalocele as the main diagnosis during the first 30 days in life.

The personal identity number, a ten-digit unique personal identity code assigned to each Swedish resident at birth, was used for correct linkages between the registers used.

### Data resources/registers

NPR was established in the 1960s and covers all in-patient care, including diagnosis and surgical procedures, since 1987. Since 2001 it also covers outpatient care but not primary care. The underreporting of data is considered very low [16–18]. From the NPR data concerning diagnosis, concurrent anomalies, syndromes and chromosomal abnormalities were collected.

MBR was founded in 1973 and covers 96–99% of all live and stillbirths with a gestational age of 22 weeks or more in Sweden [19]. The register holds prenatal, perinatal and postnatal data on both the mother and the newborn who are linked by their personal identity numbers. From MBR, we collected data concerning mode of delivery, infant diagnoses, the infants birth weight and Apgar.

The Swedish Birth Defects Register is a part of MBR and holds information on anomalies and chromosomal defects in live and stillborn children with a gestational age of 22 weeks or more. The register also holds information on fetuses after abortions induced because of fetal injuries or chromosomal defects. The Swedish Birth Defects Register was created 1963 and was adapted to the ICD-10 diagnosis system in 1999. Missing data on fetuses after induced abortions is estimated to be 30–50% [20], but considerably less for terminations after a gestational age of 22 weeks or more. From the Swedish Birth Defects Register data on anomalies and chromosomal defects concerning the children with omphalocele was gathered. Data on TOP for fetuses with a prenatal diagnosis of omphalocele were obtained from 1/1 1999 to 31/12 2016.

The Swedish Causes of Death Register (CDR) exists since 1961 and is held by the National Board of Health. All deaths in Sweden and deaths of Swedish citizens abroad are included. The cause of death is specified by the treating physician or by pathologist performing postmortem studies. From CDR, we collected data on causes of deaths within the cohort.

### Variables

The *birth prevalence* for omphalocele among lives born in Sweden was calculated for each calendar year, using information from the National Patient Register the Medical Birth Register and the Swedish Birth Defects Register. Numbers of *TOP* concerning fetuses with a diagnosis of omphalocele were calculated for each calendar year using the Medical Birth register and the Swedish Birth Defects register.

The birth prevalence for omphalocele was defined as the total number of newborns with omphalocele/10,000 live births. Data for all live births in Sweden, available from Statistics Sweden (SCB), are continuously updated and were used as denominators in the analyses [21]. *TOP rate* was calculated as the quote of all known pregnancies with omphalocele that opted for TOP.

M*ortality* was calculated based on the numbers of deaths, identified though the Swedish Register for Causes of death, divided by the number of live born with omphalocele. The causes of death were identified though the ICD-10 codes reported in the register. The mortality was also assessed within certain strata due to prevalence of associated anomalies.

To find concurrent anomalies, syndromes and chromosomal defects the registers were searched for ICD Q-diagnoses in addition to the omphalocele diagnose. The rate of *associated anomalies* was obtained by identifying the share of patients with omphalocele that also had at least one more Q-diagnose in any of the registers (NPR, MBR and CDR) and dividing it with the total number of subjects with omphalocele. In the ICD-10 system, the Q-diagnoses, Q.00–Q.99, covers all congenital anomalies, malformations and chromosomal differences. A few of the Q-diagnoses were considered irrelevant and were excluded (Q430 Meckel diverticulum, Q644 malformation of urachus, Q531, Q532, Q539 undescended testicles and Q433 Intestinal malrotation since it is always present in omphalocele malformation).

### Statistical analysis

Data are presented as means ± standard deviation (SD), median, absolute values (*n*) and frequencies (%). Incidence risk ratio (IRR) was calculated to evaluate differences in incidence over time. Poisson regression was used to investigate differences in trends, and Kaplan–Meier curves to explore differences in survival.

Significance was defined as *P* ≤ 0.05. Analyses were performed using the R software version 2.38 [22].

## Results

**Fig. 1:**
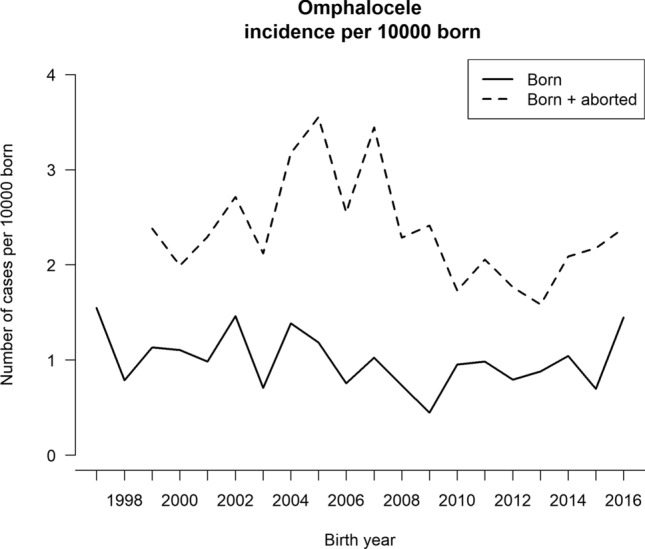
Birth prevalence and termination of pregnancy (TOP) rate for omphalocele between 1997 and 2016. The incidence for omphalocele was calculated as the number of children born with omphalocele together with the number of termination of pregnancies with diagnosed omphalocele per 10,000 born. The increase in incidence year 2004–2007 is not statistically significant

A total of 2,082,672 children were born in Sweden during the study period. Out of those, 207 had omphalocele, given the birth prevalence of 1/10,000 (Fig. 1). Between the years 1999 and 2016, a total of 449 pregnancies had a prenatal diagnosis of omphalocele, and 263 (59%) opted for termination (Fig. 1). When calculating the IRR over time, we found no statistically significant differences in incidence for omphalocele or TOP trends.

Forty-two percent of the 207 included patients were female. Mean birth weight was 2.8 kg and mean gestation age at birth was 36.5 weeks. The delivery mode was planned cesarean section (CS) in 32.9%, emergent CS in 19.9% and vaginal delivery in 31.4% of the cases. Information on delivery mode was missing in 15.8% of the cases. Results are summarized in Table 1.

**Table 1:** Birth characteristics for children born with omphalocele

*n*	207
Mean birth weight, g (SD)	2851 (± 841)
Mean gestational age, weeks (SD)	36,5 (± 3)
Mean Apgar 1 (IQR)	9 (6–9)
Mean Apgar 5 (IQR)	10 (8–10)
Mean Apgar 10 (IQR)	10 (9–10)
CS (%)	108 (52)
Sex, male (%)	120 (58)
Median follow-up time, years (IQR)	8 (2–14)

*SD* standard deviation, *IQR* interquartile range, *CS* cesarean section

Out of the 207 cases, omphalocele was an isolated diagnosis in 79 (38%) cases. We found 451 associated anomalies in 128 patients, with a range of 1–17 associated anomalies per patient. A total of 178 different associated anomalies were identified, ventricular septal defect (VSD) being the most common followed by atrial septal defect (ASD). Four children (1.9%) had trisomy 21, four (1.9%) had trisomy 18 and eight (3.9%) had trisomy 13. The rate of associated anomalies divided into different organ systems as classified in the ICD-10 system is summarized in Table 2.

**Table 2:** Associated anomalies in different organ systems

Organ system	*n*	%
CNS	15	3
ENT	39	9
Cardiovascular	176	39
Respiratory	9	2
Gastrointestinal	43	10
Genitourinary	52	12
Musculoskeletal	57	13
Chromosomal	23	5
Syndromes and other unspecified anomalies	37	8
Total	451	100

*CNS* central nervous system, *ENT* ear nose throat

**Fig. 2:**
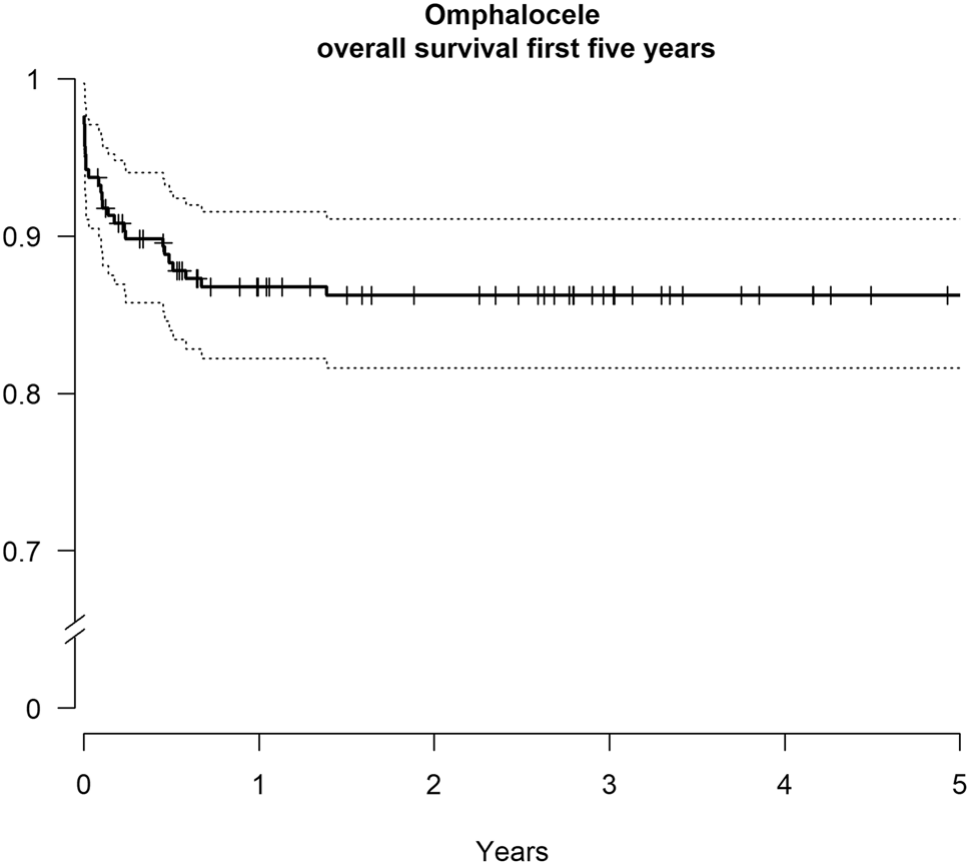
Kaplan–Meier graph showing survival over time

Six percent (*n* = 13) of the children with omphalocele died within 30 days of life and 13% (*n* = 27) before reaching 1 year. The five-year mortality was 14% (*n* = 29) and at the end of 2016, 178 patients (86%) were still alive (Fig. 2). Live born children with omphalocele and associated chromosomal anomaly had a tenfold higher risk of mortality than those with isolated omphalocele (Table 3). Among the diseased are all children born with trisomy 13 (n. 8) and trisomy 18 (n. 4). The four children born with trisomy 21 were all alive when the study was performed.

**Table 3:** Stratified mortality of children born with omphalocele

Diagnosis	*n*	n. deceased	Mortality (%)
Isolated omphalocele	79	5	6
Multiple anomalies	105	6	6
Chromosomal abnormalities	23	15	65
Total	207	26	13

## Discussion

### Key results

In this study, we found a nationwide birth prevalence for omphalocele of 1/10,000 live born over a 20-year period, with high termination rates. Over half of the parents expecting a child with prenatal diagnose of omphalocele choose to terminate the pregnancy. When pregnancy is continued, survival is high. The rate of associated anomalies in the cohort of children born with omphalocele is high: over 60% the individuals born with omphalocele have concurrent anomalies and/or chromosomal defects.

### Interpretation and generalizability

In high-income countries, with well-established health care systems and maternal care, the high rates of prenatal diagnosis contribute to the high TOP rates as shown in our setting. Previous studies have shown contradictory results regarding TOP rates with some reporting similarly high rates and others reporting lower rates of termination [1, 3, 8, 11]. Interestingly, both the birth prevalence and the TOP rates are stable and have not changed over time. The high and stable TOP rate could be partly explained by the attitude towards TOP in our cultural setting. Prenatal counseling can also be an important contributing factor in expectant parents' decisions. Conner et al. speculated on this after showing a lower TOP rate in Stockholm, where prenatal team counseling is offered, as compared to the rest of Sweden [9]. In the same study, they showed similar prevalence of associated anomalies among terminated pregnancies with omphalocele and live born children with omphalocele although only about 50% of the cases with associated anomalies amongst the live born children were prenatally diagnosed [9]. The high number of concurrent anomalies and chromosomal defects in fetuses with omphalocele probably has a significant impact on decision to opt for termination [8]. In this study, 62% of the individuals born with omphalocele had other anomalies or chromosomal defects, even though some of the anomalies were minor, e.g., protruding ears or accessory nipple. Unfortunately, we do not have further information on associated anomalies regarding the cohort that opted for TOP, but it is presumably high.

However, despite the high numbers of associated anomalies, the survival in this cohort is high, higher than previously reported [3, 4, 11]. This can be partly explained by a positive selection of cases, but also due to improvements in neonatal intensive care during the years. Still, survival is highly dependent on concurrent diagnoses. As expected, and previously reported [1, 3], the mortality is considerable higher in the group of individuals born with chromosomal anomalies as opposed to those without. Miscarriages of fetuses with omphalocele are not covered in this material.

### Strength and limitations

Sweden has well-recognized population-based registers that make it possible to perform powerful studies like this one. All data are prospectively collected and covers information on all children in Sweden; thus, we are able to describe one of the largest cohorts of patients with this rare condition. We had no loss to follow-up, which prevents selection bias. To preserve women’s privacy, the amount of data collected in the Swedish Birth Defects Register is limited. As this imply a limitation for this study, the register is still a great asset in the context.

Another limitation of the study is the impossibility to differentiate giant omphalocele from minor ones, since they share the same ICD code. Also, despite the character of the data, nationwide and covering a 20-year period, the numbers are still too low to be able to show changes in trends over the years. Even though Swedish health registries covers population health data from the 1970, prior to 1996, omphalocele and gastroschisis had a common ICD code and although they represent two different diseases with different pathology, they were often considered as one. Therefore, we had to exclude all data prior to 1997.

## Conclusion

Summarizing, the current birth prevalence in Sweden for omphalocele is low, 1 /10,000 live born, with high rates of termination for prenatally diagnosed omphalocele and high rates of associated anomalies among born children with omphalocele. However, survival of children born with omphalocele in Sweden is high.
